# Plastic plastids

**DOI:** 10.1007/s00709-023-01913-y

**Published:** 2023-12-16

**Authors:** Peter Nick

**Affiliations:** Joseph Gottlieb Kölreuter Institute for Plant Sciences, Karlsruhe Institute of Technology, Karlsruhe, Germany

To use light as source of chemical energy has been a key innovation of the cyanobacteria, and this key innovation was crucial for sustaining life on this planet. Around 1 Gya ago, a protist that had already domesticated alpha proteobacteria as functional unit able to use oxygen for respiration swallowed these cyanobacteria (Eme et al. 2014). Rather than digesting its prey, probably a member of the Gloeomargaritales, unicellular freshwater cyanobacteria (Ponce-Toledo et al. 2017), this protist stole some of the genes and, thus, the autonomy of its victim, launching a domestication process that culminated in that what we know as the extant plastid. According to the idea of symbiogenesis, first coined by the excentric Russian cell biologist Mereschkowsky (1910), the modern cell is actually a community of several organisms that have sacrificed a part of their autonomy for the sake of a more complex and more developed whole. It was Lynn Sagan (the later Lynn Margulis) who substantiated this concept in numerous cellular and molecular details (Sagan 1967) and developed this concept into a generally accepted theory. While this endosymbiont theory has successfully entered the textbooks and by now seems to be commonplace, there remain numerous fascinating questions linked to the fact that two initially independent organisms fuse to a new entity, which requires not only extensive exchange of proteins and other macromolecules but also calls for a regulatory framework sustaining the dynamic homeostasis between host and endosymbiont. Two contributions to the current issue highlight new and interesting facets of this subtle balance.

The study by MacLeod et al. (2023) is focussing on the inner membrane of plastids. Due to the endosymbiotic origin of the plastid, it must have derived from the prokaryotic surface. In fact, the contiguous peptidoglycan layer between the two membranes of the Glaucophyte plastid has been a strong argument for the endosymbiont theory. Drawing upon the great progress in the phylogenomics of early land plants, the authors ask, what has been the fate of this peptidoglycan layer during terrestrialisation. In fact, they detect seed-plant homologues of the prokaryotic penicillin-binding protein, which must be interpreted as evolutionary rudiment of the peptidoglycan layer. While the Glaucophytes still harbour a massive layer that might provide structural support, this function is unlikely to play a role for the still contiguous, but delicate peptidoglycan layer of the bryophytes, and even less for the seed plants. In bryophytes and streptophytes, this peptidoglycan layer is essential for plastid division, leading to the hypothesis that a specific member of the tubulin ancestor, FtsZ, is interacting with the peptidoglycan layer to anchor the constriction ring separating the daughter plastids (Grosche and Rensing 2017). This argument was based on patterns of co-evolution. However, now the current study presents several examples from bryophytes and streptophyte algae, where the peptidoglycan layer is present, while this specific FtsZ isoform is absent. To follow plastid division in those cases is expected to allow more insight into the function of this cyanobacterial rudiment.

In lower plants, there exist numerous examples, where plastids do not divide at all. The study by Colpo et al. (2023) investigates a very curious case for the lycophyte *Selaginella martensii*. Soon after vascular plants had conquered terrestrial habitats, they started to shape landscapes. Giant versions of extant Horsetails, called calamites, but also lycophyte clubmoss trees established luxurious forests that were the source of the fossil fuels humans exploited to drive their industrialisation. Nowadays, these lycophytes have turned into humble moss-like creatures that survive in the shade of other, more advanced trees. Their fate departed from that of other vascular plants very early, at the transition from Silur to Devon, around 415 million years ago. The main land plant lineage, the euphyllophytes, comprising the more modern ferns, the gymnosperms, and the flowering plants, populate their cells with a large number of small chloroplasts. The lycophytes instead exhibit a wide variety of chloroplast types and shapes, thus recapitulating the large variability of chloroplast morphologies found in algae and bryophytes. Within the genus *Selaginella* alone, five different types of plastids can be distinguished, whereby monoplastidy is the pre-dominant model, meaning that the cell is filled by a single giant chloroplast. These unusual chloroplasts in *Selaginella* have already inspired Gottlieb Haberlandt (1888) for a detailed study, which, by the way, contains one of the earliest descriptions of stromules. He also described the simple tissue differentiation with an upper and a lower epidermis sandwiching a spongy parenchyma. In the subgenus *Stachygynandrum*, functional differentiation includes the giant chloroplast itself. Here, the upper part of the chloroplast shows lamellae with a few thylakoids, whereas the lower part hosts thylakoid stacks resembling the well-known grana in the chloroplasts of higher plants. To describe this layering, the term bizonoplast has been coined. These zones are not fixed but subject to dynamic change. Under deep shade, the lamellae dominate, while in brighter light, the grana stacks extend. Using *S. martensii* as a model, authors demonstrate how this giant chloroplast relocates and reorganises during the day, probably as adaptation to diurnal changes in lighting conditions. Authors observed that the branches change colour during the day, whereby leaves turned pale in the evening, but were deeply green in the morning. This colour change is due to a movement of the chloroplast, which is flattening to the cell bottom under weak light, but under intense light flees to the lateral walls, such that it intercepts less light. This relocation was accompanied by thylakoid modelling during the afternoon linked with the migration of small vesicles from the chloroplast envelope to the thylakoid, thus re-establishing the plastid for the challenges of the next morning. What these vesicles contain, is not known, but a straightforward hypothesis would be that they carry metabolites that had been depleted during the morning. Interestingly, the diurnal relocation and reorganisation of the plastid is accompanied by a morphogenetic response of epidermal cells.

Both contributions highlight an astonishing complexity of plastid morphogenesis prior or briefly after terrestrialisation. This complexity seemed to be streamlined later—in direction of more numerous, but also more homogenous plastids. Thus, the gene transfer from the domesticated endosymbiont into the nucleus of the host cell was later followed by a progressive loss of plastid dynamics and morphogenetic autonomy. These seemingly exotic transitional situations show how once upon a time the plastids were endowed with a higher degree of individuality, which was later molten down into a more collectivistic and conventional behaviour, a phenomenon that seems to accompany numerous domestication processes.
